# Soldiers of Ukraine

**DOI:** 10.1016/j.lanepe.2025.101341

**Published:** 2025-06-02

**Authors:** The Lancet Regional Health – Europe

More than 3 years have passed since the full-scale Russian invasion of Ukraine began in February, 2022, a conflict that has so far mobilised over 1 million Ukrainians into military service. The toll on these soldiers—physically, psychologically, and emotionally—is immense. With relentless exposure to violence, trauma, and death, the risk of post-traumatic stress disorder (PTSD) and other mental health conditions is alarmingly high. In the absence of adequate support systems, many turn to illicit and potentially harmful substances—often the only coping mechanism available to them.

The Global Initiative Against Transnational Organized Crime has reported a surge in illicit drug use among Ukrainian soldiers since the war began particularly cannabis and synthetic substances, such as cathinones. The synthetic drug market, described as the fastest growing of all drug markets in Ukraine by the 2023 Global Organized Crime Index, is especially concerning. Synthetic substances, so-called “bath salts”, are cheap, easy to produce, and linked to severe health effects, including extreme behavioural changes, hyperthermia, cardiac arrest, depression, and suicide. If harmful drug use and its underlying causes are not addressed, they are likely to escalate and result in further tragic outcomes, including gender-based violence, family breakdown, and intergenerational trauma. The consequences of the war are far-reaching and require a health system equipped to respond to the physical and psychological toll of prolonged conflict.

Personal testimonies reveal how this crisis is affecting families and communities. A wife of a currently serving soldier described how, during his last home leave, her husband isolated himself and drank constantly—behaviour mirrored by others in her circle, with some turning to drugs and gambling. Another wife of a demobilised veteran shared how addiction, emotional distance, and anger have made reintegration into family life nearly impossible. These accounts reflect a growing public health emergency where families are left to cope without professional support.

Recognising the need for more tools to help individuals to cope with the stress and trauma from the war, the Ukrainian Parliament approved the use of cannabis for medical purposes in December, 2023, and established a working group to assess the effectiveness of psychedelic-assisted therapies such as 3,4-methylenedioxymethamphetamine on PTSD. Medical cannabis was subsequently legalised in August, 2024, and can be prescribed for PTSD—subject to the decision of a medical advisory commission.

While remaining open to novel therapies is important, addressing the structural challenges within Ukraine's health-care system is crucial. What Ukraine urgently needs is a substantial scale-up of its overstretched health workforce and infrastructure, including community-based services that collaborate with local actors such as veteran organisations, to care for soldiers and veterans, with sustained international investment that goes beyond military defence aid.

The current emphasis on weapons and strategy risks overlooking their wellbeing. Systematic screening for PTSD should be implemented within the military and when personnel transition to civilian life, alongside integrated mental health and substance use treatment pathways tailored to the complex needs of this population.

Military zero-tolerance policies on illicit drug use, which can result in punishment, loss of income, or denial of posthumous compensation, discourage disclosure and treatment. Destigmatising mental illness is essential, particularly as many soldiers avoid seeking help. “Addiction is not a weakness”, said one psychologist who has worked with veterans since 2018. “It requires understanding, compassion, and a comprehensive professional response.” Without trauma-informed rehabilitation, these individuals are likely to face long-term struggles with reintegration, family life, and employment.

Caring for soldiers’ mental, psychological, and physical health is not only a clinical responsibility, but also a moral and political one, and a necessary step towards long-term social stability. Their wellbeing must be central to any conflict response. As Ukraine continues to bear the burden of war, international donors and institutions must broaden their aid strategies beyond defence to prioritise mental health and rehabilitation, including the development of trauma rehabilitation centres. Failing to confront these issues risks pushing Ukraine into a serious long-term public health crisis. Meeting these needs now is crucial to lay the groundwork for a healthier, more resilient post-war Ukraine.

